# Improved Bacterial Single-Cell RNA-Seq through Automated MATQ-Seq and Cas9-Based Removal of rRNA Reads

**DOI:** 10.1128/mbio.03557-22

**Published:** 2023-03-07

**Authors:** Christina Homberger, Regan J. Hayward, Lars Barquist, Jörg Vogel

**Affiliations:** a Institute of Molecular Infection Biology (IMIB), University of Würzburg, Würzburg, Germany; b Helmholtz Institute for RNA-based Infection Research (HIRI), Helmholtz Centre for Infection Research (HZI), Würzburg, Germany; c Faculty of Medicine, University of Würzburg, Würzburg, Germany; Massachusetts Institute of Technology

**Keywords:** MATQ-seq, single-cell RNA-seq, *Salmonella enterica*, rRNA depletion, gene expression heterogeneity, DASH, Cas9

## Abstract

Bulk RNA sequencing technologies have provided invaluable insights into host and bacterial gene expression and associated regulatory networks. Nevertheless, the majority of these approaches report average expression across cell populations, hiding the true underlying expression patterns that are often heterogeneous in nature. Due to technical advances, single-cell transcriptomics in bacteria has recently become reality, allowing exploration of these heterogeneous populations, which are often the result of environmental changes and stressors. In this work, we have improved our previously published bacterial single-cell RNA sequencing (scRNA-seq) protocol that is based on multiple annealing and deoxycytidine (dC) tailing-based quantitative scRNA-seq (MATQ-seq), achieving a higher throughput through the integration of automation. We also selected a more efficient reverse transcriptase, which led to reduced cell loss and higher workflow robustness. Moreover, we successfully implemented a Cas9-based rRNA depletion protocol into the MATQ-seq workflow. Applying our improved protocol on a large set of single Salmonella cells sampled over different growth conditions revealed improved gene coverage and a higher gene detection limit compared to our original protocol and allowed us to detect the expression of small regulatory RNAs, such as GcvB or CsrB at a single-cell level. In addition, we confirmed previously described phenotypic heterogeneity in Salmonella in regard to expression of pathogenicity-associated genes. Overall, the low percentage of cell loss and high gene detection limit makes the improved MATQ-seq protocol particularly well suited for studies with limited input material, such as analysis of small bacterial populations in host niches or intracellular bacteria.

## INTRODUCTION

Until now, bacterial transcriptome studies have mainly relied on bulk RNA sequencing (RNA-seq) (1). This approach provides averaged gene expression values across an entire cell population and therefore does not allow conclusions regarding transcriptional heterogeneity between individual bacteria. Yet, such phenotypic heterogeneity is a common microbial phenomenon (2). It is important for bacterial survival strategies such as bet hedging, which allows fast adaptations to changing environments (3, 4), or biofilm formation, in which individual cells take on highly specific roles within a community (5).

Dating to 2009, pioneering work established single-cell RNA-seq (scRNA-seq) in eukaryotes (6). While this field rapidly evolved (7), the development of scRNA-seq in bacteria was slow to progress due to several challenges (8). Prokaryotic cells are much smaller than eukaryotic cells, leading to less input material per cell. Single bacteria contain RNA in the femtogram range (9) and the average mRNA copy number is low, at only 0.4 copies/cell (10). Further challenges include efficient cell lysis, which is hampered by the bacterial cell wall, and capture of nonpolyadenylated bacterial transcripts. These differences prevent a direct adaptation of most eukaryotic single-cell transcriptomic workflows.

Nevertheless, thanks to technical advances, bacterial single-cell transcriptomics has recently become a reality (8). Three general types of approaches are currently available. Bacterial multiple annealing and deoxycytidine (dC) tailing-based quantitative scRNA-seq (MATQ-seq) (11) is a workflow originally developed for eukaryotes (12) that relies on cell isolation by fluorescence-activated cell sorting (FACS) and random priming of cellular transcripts. A second type, also previously established for eukaryotes and termed split-pool ligation transcriptomics sequencing (SPLiT-seq) (13), is based on combinatorial barcoding. It was adapted for bacterial scRNA-seq in two independent studies introducing the so-called prokaryotic expression profiling by tagging RNA *in situ* and sequencing (PETRI-seq) and microbial SPLiT-seq (microSPLiT) protocols (14, 15). In comparison to MATQ-seq, bacterial split-pool barcoding workflows enable the analysis of thousands of cells instead of a few hundred, offsetting the lower transcript capture rate and higher rate of cell loss in these protocols. The third type is a microscopy- and probe-based approach that does not employ RNA-seq. It is called parallel sequential fluorescence *in situ* hybridization (par-seqFISH) and allows spatial transcriptomics on the level of single bacteria (16).

Despite these recent advances, challenges remain. These include a high frequency of cell loss and problems with robustness, coverage and prevalence of redundant rRNA. In addition, short transcripts, such as small regulatory RNAs (sRNAs), show poor coverage or are not measurable at all. Importantly, transcript detection is currently limited to ~200 genes per single cell, which is far below the average bacterial transcriptome. We reasoned that targeted improvements of our previous MATQ-seq protocol would address some of these challenges.

In this work, we present the next version of bacterial MATQ-seq. While the original protocol (11) has a high transcript capture rate, including low-abundance transcripts, it is also limited in throughput and robustness. Through the integration of automation, we have now achieved increased cell throughput. In addition, we improved robustness through the selection of a more efficient reverse transcriptase (RT), which also led to a reduced transcript dropout rate. Finally, given that in our previous protocol the vast majority of reads represented rRNA, we integrated a Cas9-mediated targeted rRNA depletion protocol, called depletion of abundant sequences by hybridization (DASH) (17). This allowed us to obtain more gene expression information per single cell with decreased sequencing costs.

## RESULTS

### Automation of the MATQ-seq workflow achieves higher throughput.

**FIG 1:**
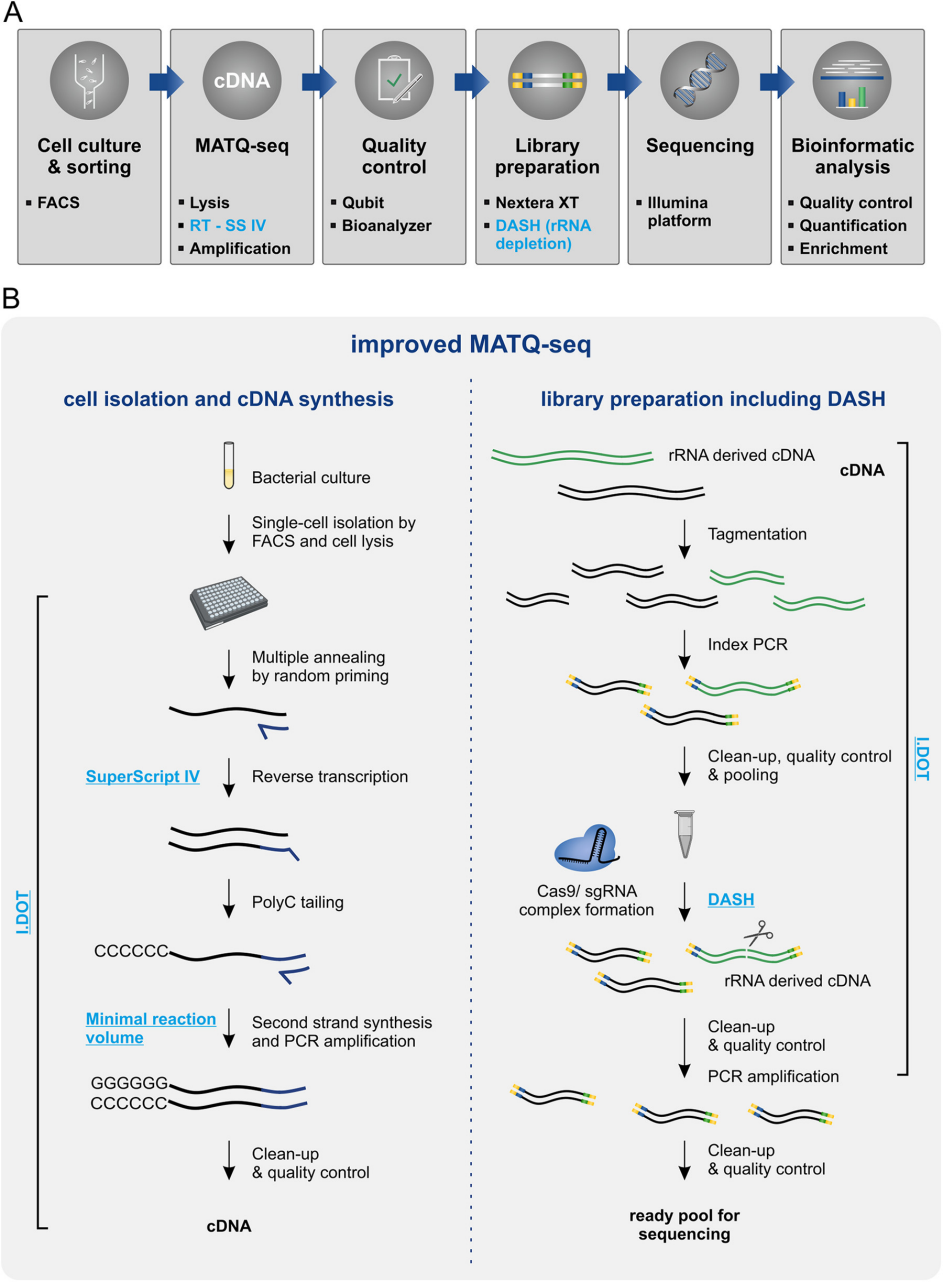
Improved MATQ-seq workflow for bacterial single-cell RNA-seq. (A) Overview of bacterial scRNA-seq pipeline including major steps from cell culture to bioinformatic analysis. Changes from the previous MATQ-seq protocol are highlighted in blue. (B) Detailed workflow of the MATQ-seq protocol separated into two main steps: cell isolation and cDNA synthesis (left) and library preparation including DASH for rRNA depletion (right). Major improvements are highlighted in blue, including the use of SuperScript IV (SS IV) for reverse transcription, reaction optimization, and integration of DASH into the library preparation. All pipetting steps were automated using the I.DOT dispensing robot, with the exception of all cleanup and quality control steps.

Initially, we aimed to increase cell throughput and read quality by integrating automation and by refining our analysis pipelines. In order to enable direct comparison with previous data, we performed all experiments with Salmonella enterica serovar Typhimurium. Within the MATQ-seq protocol, library preparation and quality control consist of a series of different labor-intensive pipetting steps. We implemented a user-friendly and highly flexible automation process by establishing protocols for all pipetting steps on the I.DOT dispensing robot (Dispendix), with the exception of cleanup and quality control steps (Fig. 1). This decreased turnaround times and the amount of consumables needed. Concurrently, automation increased sample throughput.

For cDNA analysis and quality control, we integrated the high-throughput Qubit Flex fluorometer for a precise and fast procedure. To facilitate sample processing, we applied a miniaturization step for the final PCR volume by skipping the splitting step of the PCR implemented in the original MATQ-seq protocol (12). This allowed processing of up to 96 single cells per MATQ-seq passage, compared to a maximum of 24 cells in the previous protocol, and decreased the overall processing time from about 10 to 8 h. More importantly, the hands-on time was reduced from about 6 to 3 h. Finally, we updated our data processing and analysis pipeline to improve data quality by implementing a better trimming approach, alignment, normalization, and identification of outliers (see Materials and Methods for details). Overall, all these steps discussed above led to higher cell throughput, improved accuracy, and higher read qualities, as described in more detail below.

### Optimized reverse transcription leads to higher robustness and reduced cell loss.

Reverse transcription is crucial for RNA conversion and thus greatly affects the robustness of the scRNA-seq protocol and the detection of low-abundance transcripts. In our previous study, we used SuperScript III (SS III) for the reverse transcription step (11). In the meantime, RTs with properties that promised to improve reverse transcription efficiency were reported (18–20). Therefore, we systematically tested SuperScript IV (SS IV), an optimized RT based on SS III with improved thermostability, robustness, and processivity; TGIRT and PrimeScript, two RTs that allow reverse transcription of GC-rich regions and highly structured RNAs; and Maxima H Minus and SMARTScribe, two highly efficient RTs with a high processivity and the capability to convert RNA transcripts up to 20 or 14.7 kb in length. For the analysis of these five different RTs we used fluorometer (Qubit) as well as high-resolution gel electrophoresis (Bioanalyzer) systems to assess cDNA yield and integrity.

**FIG 2:**
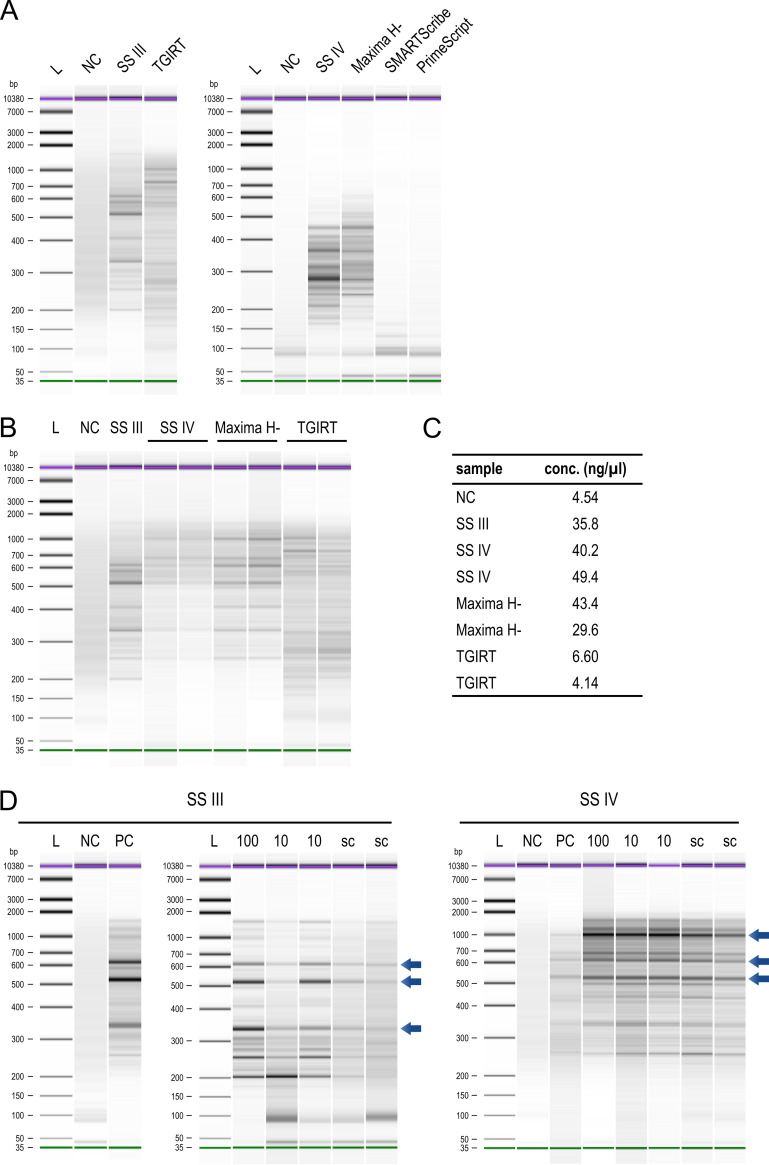
Selection of alternative reverse transcriptase. Shown are Bioanalyzer profiles of cDNA processed by MATQ-seq using different reverse transcriptases. (A) Comparison of five different reverse transcriptases with SS III. Sample input was 50 ng of total RNA for all conditions. (B) Further validation of the three RTs SS IV, Maxima H Minus (Maxima H-), and TGIRT after initial selection and subsequent buffer optimization compared to panel A. All assays were performed with a spike-in of 50 pg of total RNA. (C) cDNA concentrations of samples in panel B measured with a Qubit fluorometer. (D) Comparison of cDNA profiles obtained with SS III (left) and SS IV (right). Each profile represents the cDNA prepared from either a single cell (sc) or 10-sorted or 100-sorted cells. Sorted cell conditions served as a control to evaluate cDNA integrity obtained from a single cell. The positive control was performed with a spike-in of 50 pg of total RNA. Characteristic bands are indicated with blue arrows. L, ladder; NC, negative control; PC, positive control.

Initially, we used total RNA as a spike-in to evaluate the compatibility of the RTs with the MATQ-seq workflow (Fig. 2A). Of note, reverse transcription during MATQ-seq is performed with temperature gradients, which might interfere with RT efficiency. Indeed, SMARTScribe and PrimeScript showed low efficiency and were excluded from further validation. For the remaining three RTs, we adapted buffer conditions to the manufacturer’s recommendation. This yielded larger fragments in Bioanalyzer profiles, especially for SS IV and Maxima H Minus (Fig. 2B). Qubit measurements of cDNA obtained from spike-in tests using TGIRT showed low yield insufficient for further library preparation, ruling out this RT for further use (Fig. 2C).

Next, we assessed the performance of SS IV and Maxima H Minus on sorted cells. Whereas the two RTs showed similar results for 50 and 10 sorted cells, Maxima H Minus was less efficient at the single-cell level (see Fig. S1 in the supplemental material). In a direct comparison of SS IV to SS III (Fig. 2D), SS IV showed a higher reproducibility, indicated by a pattern of characteristic bands and an increase in cDNA yield at the single-cell level. Based on these results and a lower cell loss overall, we implemented SS IV in the MATQ-seq protocol. Of note, due to the implementation of automation and the improvements in reverse transcription, only ~10% of all cells were lost during wet-lab processing (MATQ-seq and library preparation) and/or excluded by bioinformatic filtering.

10.1128/mbio.03557-22.1 FIG S1 Validation of reverse transcriptases. Shown is a comparison of cDNA profiles obtained using SuperScript III (SS III) and SS IV (left) and Maxima H Minus (Maxima H-) (right). For SS IV and Maxima H-, each profile represents the cDNA prepared from either a single cell (sc) or 10-sorted or 50-sorted cells. L, ladder; NC, negative control; PC, positive control. Download FIG S1, TIF file, 0.2 MB. Copyright © 2023 Homberger et al. 2023 Homberger et al. https://creativecommons.org/licenses/by/4.0/ This content is distributed under the terms of the Creative Commons Attribution 4.0 International license.

### rRNA depletion substantially increases non-rRNA reads.

In order to reduce rRNA-derived reads, we applied an rRNA depletion step using DASH, a Cas9-mediated cleavage protocol originally developed in eukaryotes that can be implemented at the cDNA level (17). Specifically, a pool of single guide RNAs (sgRNAs) targeting rRNA-derived cDNA is provided together with Cas9, causing targeted cDNA cleavage. DASH had already been shown to work efficiently in low-input bacterial samples (>1 ng of total input RNA) (21) as well as at a single-cell level in eukaryotes (22).

In order to optimize DASH conditions to our protocol, we tested five molar ratios of Cas9 to sgRNAs in the range from 1:2 to 1:50 and compared the percentages of mapped rRNA reads (Fig. S2). A ratio of 1:2 was the most efficient and led to an rRNA depletion of 75%. These results are consistent with previously described DASH protocols for rRNA depletion in bacterial bulk samples, reporting depletion efficiencies in the range of 30 to 60% for Salmonella (21).

10.1128/mbio.03557-22.2 FIG S2 Comparison of different ratios of Cas9 and sgRNA for DASH. Shown is the RNA class distribution obtained with different Cas9/sgRNA ratios. Per ratio, three single cells were processed by MATQ-seq including DASH under anaerobic shock conditions. The RNA classes are shown as a percentage of the mean of the mapped reads (*n* = 3 single cells). Numbers below the graph represent the relative molar access of Cas9 and sgRNA over a single cDNA fragment. CDS, coding sequence; ncRNA, noncoding RNA. Download FIG S2, TIF file, 0.1 MB. Copyright © 2023 Homberger et al. 2023 Homberger et al. https://creativecommons.org/licenses/by/4.0/ This content is distributed under the terms of the Creative Commons Attribution 4.0 International license.

**FIG 3:**
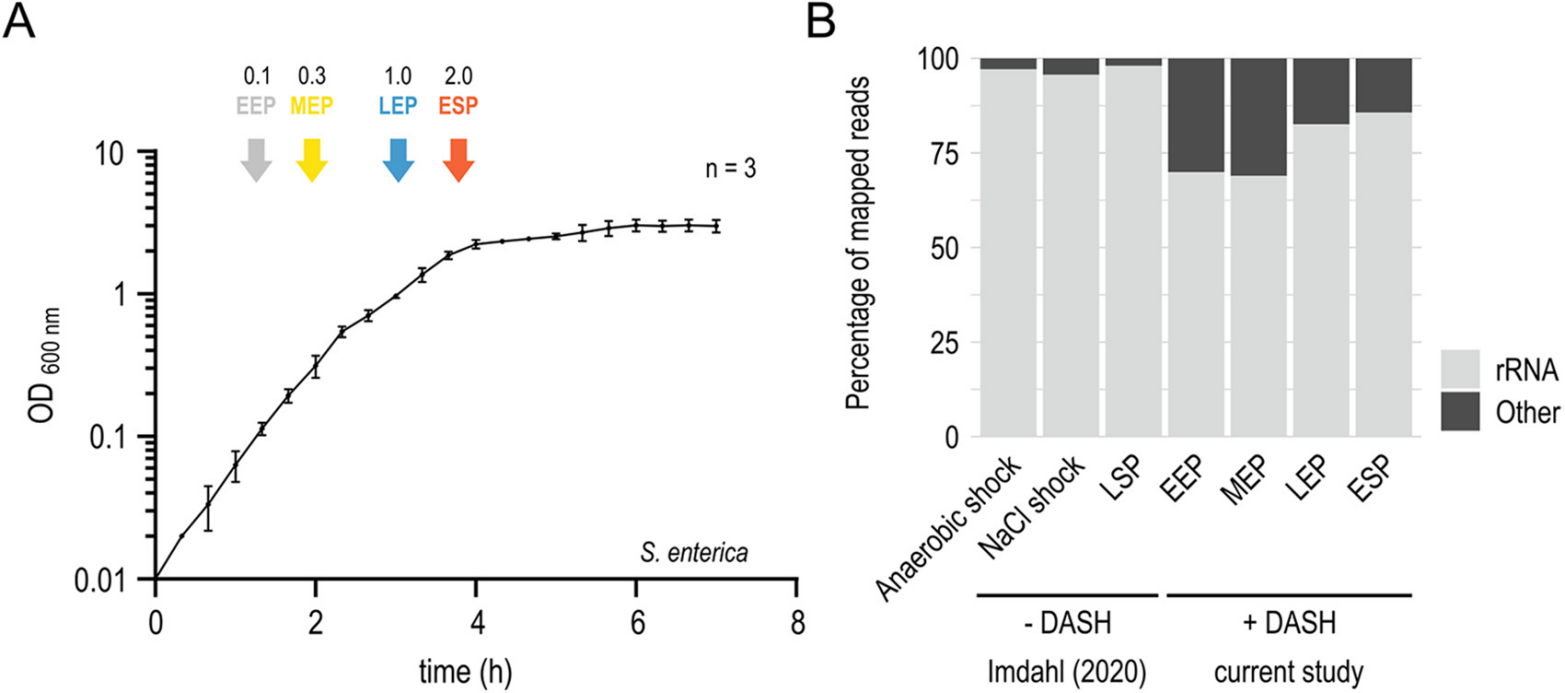
Experimental design and RNA class distribution. (A) Growth curve of Salmonella in LB medium, with colored arrows indicating the four sampling points for scRNA-seq experiments. Data are displayed as means ± standard deviation (SD) of three independent experiments. (B) Representation of RNA class distribution comparing both protocols under different growth conditions. See Table S1A for detailed information on the prevalence of each RNA class. EEP, early exponential phase; MEP, mid-exponential phase; LEP, late exponential phase; ESP, early stationary phase; LSP, late-stationary phase.

To evaluate rRNA depletion efficiency on a larger scale and across growth conditions, we applied it to Salmonella in early exponential (EEP), mid-exponential (MEP), late exponential (LEP), and early stationary (ESP) phases (Fig. 3A). Per condition, 96 single cells were processed by MATQ-seq. DASH is performed after tagmentation and introduction of full adapter index sequences by index PCR. Without this preamplification step, we were not able to recover enough cDNA after DASH. Since indices that allow cell identification are introduced during index PCR, samples can be pooled before DASH, thereby reducing the number of DASH reactions (Fig. 1 and Fig. S3).

10.1128/mbio.03557-22.3 FIG S3 Implementation of sc-DASH workflow. Shown is a detailed overview of the integration of a single-cell-compatible DASH workflow into the Nextera XT library preparation protocol. The right panel lists the most important adaptations required compared to the published bulk-DASH pipeline (G. Prezza, T. Heckel, S. Dietrich, C. Homberger, et al., RNA 26:1069–1078, 2020, https://doi.org/10.1261/rna.075945.120). Download FIG S3, TIF file, 0.3 MB. Copyright © 2023 Homberger et al. 2023 Homberger et al. https://creativecommons.org/licenses/by/4.0/ This content is distributed under the terms of the Creative Commons Attribution 4.0 International license.

10.1128/mbio.03557-22.10 TABLE S1 (A) Proportion of mapped reads to gene biotypes; (B) genome coverage for new cells; (C) genome coverage for previous cells; (D) sRNAs—TPM expression; (E) list of Salmonella strains; (F) list of oligonucleotides; (G) adapter and primer sequences used with BBDuk. Download Table S1, XLSX file, 0.3 MB. Copyright © 2023 Homberger et al. 2023 Homberger et al. https://creativecommons.org/licenses/by/4.0/ This content is distributed under the terms of the Creative Commons Attribution 4.0 International license.

The newly generated data set showed a much higher percentage of non-rRNA reads independent of growth condition (Fig. 3B) than did the data obtained using our original MATQ-seq protocol (11). Importantly, in contrast to single-cell libraries that were not treated with DASH, we detected up to a 10-fold-higher percentage of reads mapped to coding sequences (CDSs), sRNAs, tRNAs, and untranslated regions (UTRs), indicating successful elimination of rRNA-derived cDNA upon Cas9-mediated cleavage. Of note, the overall distribution of all other RNA classes did not vary substantially among growth conditions or between the two protocols, suggesting no major Cas9 off-target effect leading to unwanted cleavage of libraries (Table S1A). Compared to our initial test experiments (Fig. S2) using cDNA from sorted single cells grown under anaerobic shock conditions, the rRNA depletion efficiency was lower in this larger-scale experiment, although we used the same Cas9/sgRNA ratio. This might be due to an additional pooling step that could have saturated the Cas9 enzyme or to differences in commercial Cas9 batches that were used. Nevertheless, the DASH step still decreased rRNA reads up to ~30%.

The successful implementation of DASH required several adjustments to facilitate compatibility with the scRNA-seq workflow. Specifically, pre- and postamplification cycles were optimized to ensure enough yield and, at the same time, to prevent overamplification. We found that heat inactivation of proteinase K used in published DASH protocols was highly inefficient in our experimental settings. As a result, the downstream PCR was negatively affected by proteinase K and rRNA-depleted libraries were not amplified adequately. Inactivation by phenylmethylsulfonyl fluoride (PMSF) led to higher efficiency and allowed PCR amplification of the final library pool (Fig. S3). Another important adjustment was the cleanup procedure required to remove PCR reagents, especially primer dimers from PCR products. The column-based purification used in published protocols was not suitable due to high sample loss. Instead, we used magnetic beads for the cleanup, which allowed us to purify low-input PCR samples with minimal sample loss. The ratio of magnetic beads and PCR product was adjusted to 1:1 (vol/vol), thereby ensuring capture of short fragments, including ones derived from short transcripts, such as sRNAs (Fig. S2).

### Improved MATQ-seq provides better gene coverage and shifts the gene detection limits.

**FIG 4:**
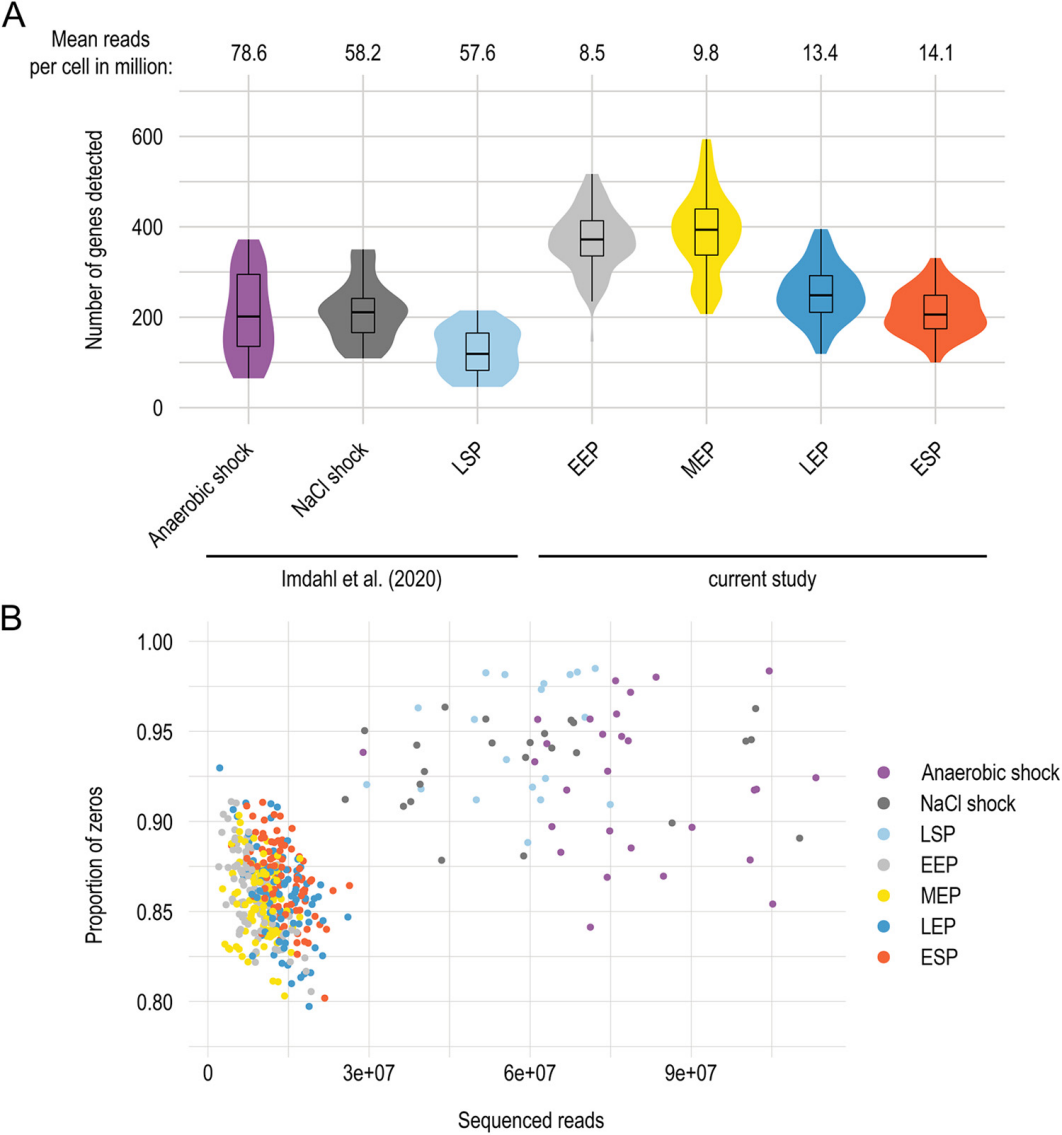
Gene detection limit and robustness of improved MATQ-seq workflow. (A) Overlaid violin and boxplots showing the median, quartiles, and distribution for the numbers of detected genes per condition. Mean numbers of reads per single cell are indicated at the top. (B) Proportion of genes with no assigned reads (zeros) per single cell compared to the number of sequenced reads, with each color-coded dot representing a single cell.

Due to the improvements we implemented, we were able to reduce the sequencing depth compared to our original MATQ-seq protocol (11) and still detect more genes on average across the four growth conditions than previous data: 307 versus 185 genes, respectively (Fig. 4A). We detected the highest number of genes in cells sampled in EEP and MEP, in which more than 375 genes were detected on average (Fig. 4A). This is in line with expectations, as the mRNA level/cell has previously been observed to increase during exponential growth (23). Despite reducing the sequencing depth ~6-fold, we achieved increased sensitivity of gene detection as the proportion of genes with no assigned reads (“zeros”) across cells was reduced (Fig. 4B). This is directly related to the proportion of genes we detected across cells genome-wide, which increased to 95% (Fig. S4), but also genome coverage, which increased from 3.0× to 4.8× (Tables S1B and C).

10.1128/mbio.03557-22.4 FIG S4 Percent genes detected. The number of detected genes across all cells per condition for both MATQ-seq data sets is shown. Download FIG S4, TIF file, 0.1 MB. Copyright © 2023 Homberger et al. 2023 Homberger et al. https://creativecommons.org/licenses/by/4.0/ This content is distributed under the terms of the Creative Commons Attribution 4.0 International license.

We also wanted to assess how well our scRNA-seq data corresponded to condition-matching bulk RNA-seq data. Therefore, we generated pseudo-bulk data by summing gene expression across all cells per condition and compared them against bulk RNA-seq data of samples taken from the same culture as the sorted single cells. To assess possible biases introduced by DASH, we performed bulk RNA-seq library preparation using a standard affinity probe-based rRNA depletion method. Using three bulk RNA-seq replicates, we obtained higher correlations than previously observed (11), confirming a closer resemblance to bulk RNA-seq data (Fig. S5).

10.1128/mbio.03557-22.5 FIG S5 Density plots and the corresponding correlations between scRNA-seq data and bulk RNA-seq data of different growth conditions. Spearman’s correlation between pseudo-bulk (counts summed across cells per condition) and bulk RNA-seq data for all four growth conditions is shown; colored squares represent the amount of overlapping genes. Three bulk replicates were used, and the associated correlations (r1 - r3) are shown. EEP, early exponential phase; MEP, mid-exponential phase; LEP, late exponential phase; ESP, early stationary phase. Download FIG S5, TIF file, 0.6 MB. Copyright © 2023 Homberger et al. 2023 Homberger et al. https://creativecommons.org/licenses/by/4.0/ This content is distributed under the terms of the Creative Commons Attribution 4.0 International license.

### MATQ-seq enables detection of small regulatory RNAs.

**FIG 5:**
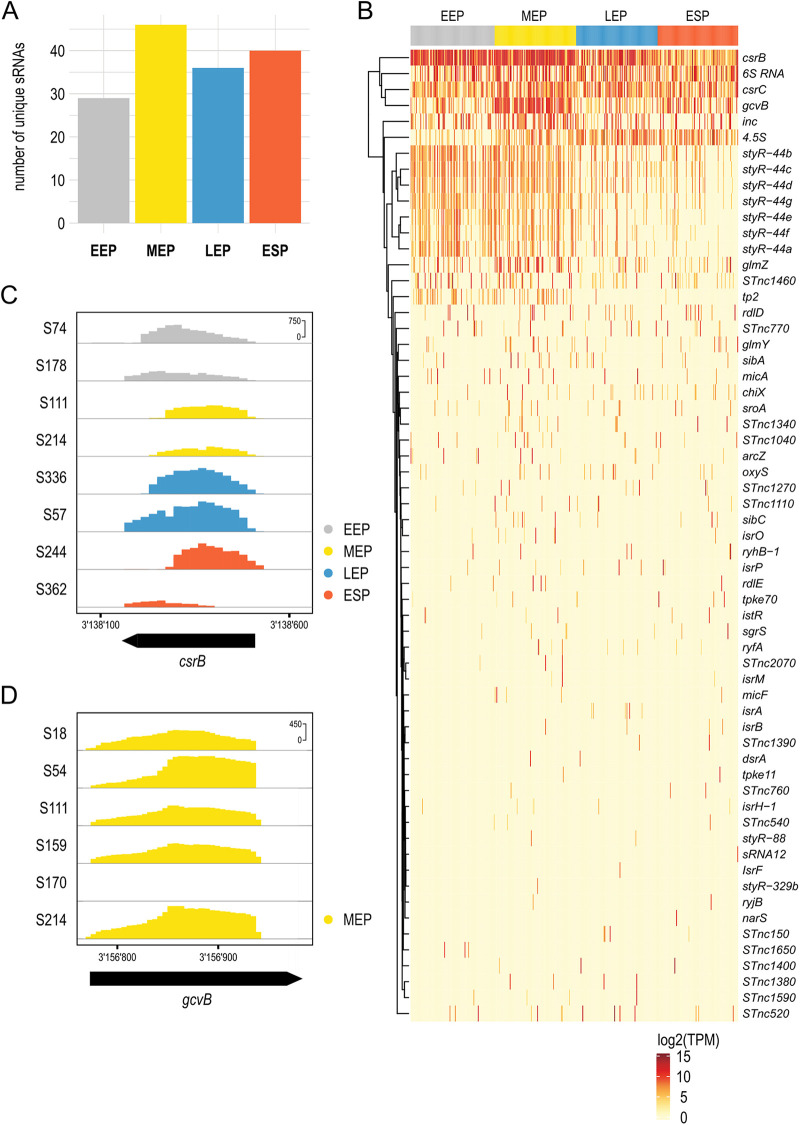
Small regulatory RNA regulation at a single-cell level. (A) Representation of unique sRNAs identified under each growth condition. (B) Heat map showing prevalence and distribution of the most abundant sRNAs under different growth conditions. (C) Coverage plot and read densities of sRNA CsrB in eight selected single cells (indicated by sample number). (D) Coverage plot and read densities of sRNA GcvB in MEP in six selected single cells (indicated by sample number).

sRNAs play a major role in bacterial gene regulation and are important during stress responses and virulence (24, 25). However, short transcripts, like sRNAs, are notoriously difficult to detect at the single-cell level due to inefficient recovery during the scRNA-seq workflow. In addition, cleanup procedures are required to remove primer dimers, but these bear risk to also target sRNAs which can be similar in size. Nevertheless, the use of magnetic beads at high ratio (1:1 [vol/vol]) that we adapted in MATQ-seq for this purpose improved the recovery of smaller fragments. As a result, we were able to detect a large number of sRNAs across the different growth conditions (Table S1D) and between 28 to 46 unique sRNAs per condition (Fig. 5A). We observed a high variability in the overall prevalence and expression level of sRNAs across conditions but also within the same growth condition (Fig. 5B). For example, the sRNAs CsrB and CsrC, known regulators of the global RNA-binding protein CsrA (26), were among the most prevalent sRNAs detected and showed highly variable expression across different growth conditions (Fig. 5B). The Csr complex constitutes one of the key regulatory systems for virulence, stress responses, motility, and biofilm formation in Salmonella (27). In accordance with our data, condition-dependent differences in expression levels of both sRNAs have previously been described (28). The technical feasibility to detect sRNAs at a single-cell level was substantiated by *csrB* reads that only covered its transcribed region, indicating that coverage does not arise from processing artifacts. Though uneven mapping was observed among different single cells (Fig. 5C), a broad coverage of *csrB* transcripts suggests robust detection of sRNAs by MATQ-seq.

As an additional example, the sRNA GcvB, whose regulon mainly includes enzymes involved in amino acid biosynthesis and transporters, was highly expressed in the majority of cells in the MEP phase (Fig. 5B). Nevertheless, we also observed cells that did not express *gcvB* (Fig. 5D). This finding is in accordance with earlier studies investigating *gcvB* expression in bulk samples; however, we did not observe a complete absence of GcvB in the stationary phase as previously seen (29). Instead, we detected a highly variable expression of GcvB in the ESP. Therefore, despite low (or nondetectable) expression at the whole-population level, some cells still express GcvB, indicating heterogeneity across the cell population. Overall, these examples show that our improved MATQ-seq protocol enables detection of sRNAs at the single-cell level, reflecting expression patterns that have been previously reported using bulk RNA-seq data sets.

### Variability in Salmonella gene expression over different growth conditions.

**FIG 6:**
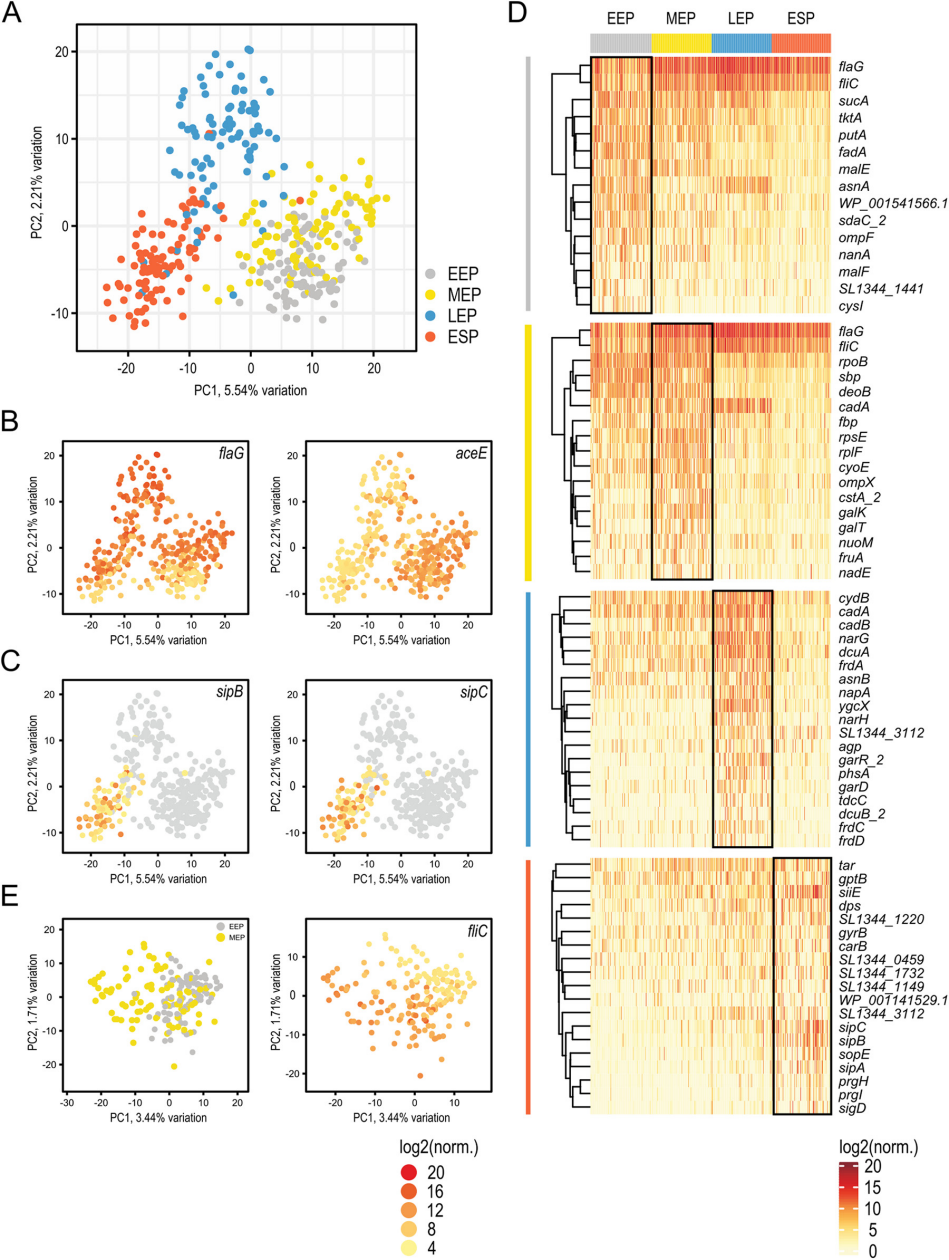
Cluster identification and analysis of highly variable genes detected at a single-cell level. (A) Principal component analysis (PCA) of all analyzed cells across the four growth conditions. (B) Overlay of the expression of genes contributing to the separation of the three main clusters in panel A. (C) Expression of Salmonella pathogenicity genes *sipB* and *sipC* within ESP. (D) Heat map of the gene expression level of the top 1% of highly variable genes detected for each growth condition. (E) (Left) PCA analysis of cells from EEP and MEP. (Right) Overlay of expression of the flagellar gene *fliC* on the PCA blot shown on the left.

Visualization of all analyzed cells using principal component analysis (PCA) showed variation in gene expression over the different growth phases in Salmonella, as expected (Fig. 6A). EEP and MEP cells clustered together, in line with rapid cellular proliferation during these growth phases, which necessitates high gene expression (23). LEP and ESP were more distinct, reflecting the onset of nutrient starvation and a less active cellular state (30). Genes that drive the separation of these three main clusters are involved with growth-related processes and have previously been described (31). They include genes encoding components of flagella (*flaG*, also known as *flhB*, and *fliC*), lipid metabolism (*fadB*), glycolysis (*aceE*), and others (Fig. S6). Overlaying the gene expression across all cells in the same PCA plot helps visualize these expression patterns across and within conditions (Fig. 6B and C).

10.1128/mbio.03557-22.6 FIG S6 Drivers of heterogeneity. Results for principal component analysis based on Fig. 6A in the main text that includes the genes contributing the most amount of variation to separate the clusters across principal components 1 (*x* axis) and 2 (*y* axis) are shown. Download FIG S6, TIF file, 0.3 MB. Copyright © 2023 Homberger et al. 2023 Homberger et al. https://creativecommons.org/licenses/by/4.0/ This content is distributed under the terms of the Creative Commons Attribution 4.0 International license.

We extracted the most highly variable genes (HVG) within each condition (Fig. 6D), highlighting their heterogeneous expression within a cell population. Of particular interest was the ESP, because we observed numerous genes related to Salmonella pathogenicity (*tar*, *siiE*, *sipA*, *sipB*, *sipC*, *prgH*, and *prgI*) that appeared to be associated with specific groups of cells (Fig. 6D). To explore this further, we focused on genes expressed from Salmonella pathogenicity islands (SPI) 1, 2, and 4 (Fig. S7). We observed three subpopulations based upon a group of ~7 SPI genes, exhibiting very low or no expression (Fig. S7, middle), low expression (left), and high expression (right). Bulk RNA-seq studies have shown that SPI genes are more highly expressed during ESP than under earlier growth conditions (31). Variation in expression of selected genes has previously also been reported at the single-cell level (32). In this study, by examining a larger set of SPI genes, we observed that a small subset of these genes mediates clustering of the cells into different populations.

10.1128/mbio.03557-22.7 FIG S7 Heat map of Salmonella pathogenicity island (SPI) genes. Shown is a heat map with cells from ESP showing log_2_ (transcripts per million) gene expression of Salmonella pathogenicity-encoding genes. Cells are clustered gene-wise (horizontal) and cell-wise (vertical), revealing three different subgroups. The intensity of the blue color code correlates with gene expression level. Download FIG S7, TIF file, 0.4 MB. Copyright © 2023 Homberger et al. 2023 Homberger et al. https://creativecommons.org/licenses/by/4.0/ This content is distributed under the terms of the Creative Commons Attribution 4.0 International license.

To further analyze early growth conditions, we visualized only EEP and MEP cells with PCA (Fig. 6E). Due to the limited variation from each principal component, the cells remained closely clustered, further highlighting their similarities. Returning to the HVG, the two flagellar genes *flaG* and *fliC* showed particularly high variability in both growth phases (Fig. 6D). Both genes encode proteins of the core flagellum, and their expression has a direct impact on cell motility (33). Overlaying the expression of *fliC* suggests two distinct subpopulations of cells, including both EEP and MEP cells (Fig. 6E, right). To visualize if additional flagellar genes show the same pattern, we generated heat maps containing flagellum-expressing genes (Fig. S8). While we did not detect distinct subpopulations, we did see a gradient in flagellar gene expression, with a wide range of expression of flagellar genes within each growth phase. This observation is in accordance with previous findings describing heterogeneous expression of *fliC* in Salmonella directly associated with cell motility and the potential to evade host inflammatory responses (2, 34).

10.1128/mbio.03557-22.8 FIG S8 Flagellar genes. Shown are heat maps with cells from EEP (left panel) and MEP (right panel) expressing flagellar genes. Cells within same growth condition are clustered gene-wise (horizontal) and cell-wise (vertical). The intensity of the blue color code correlates with gene expression level. Download FIG S8, TIF file, 0.7 MB. Copyright © 2023 Homberger et al. 2023 Homberger et al. https://creativecommons.org/licenses/by/4.0/ This content is distributed under the terms of the Creative Commons Attribution 4.0 International license.

### Effects of sequencing depth.

The high number of sequenced reads per cell that we obtained allowed us to explore gene detection limits per cell and how cell clustering is affected as sequence depth decreases. For both issues, we simulated scRNA-seq data across all cells for each condition separately (see Materials and Methods), querying different sequencing depths. Independent of the experimental condition, we reached saturation of the number of detected genes per cell at around 5 million reads (including rRNA-derived reads [Fig. S9A]). This saturation analysis will assist future studies using MATQ-seq to find a balance between sequencing depth, the number of cells to analyze, and the associated costs.

10.1128/mbio.03557-22.9 FIG S9 Saturation and downsampling PCA with detected genes. (A) Reads per cell from simulated scRNA-seq data plotted against the number of detected genes. The right panel shows a zoomed-in view of the boxed area. (B) Series of PCA plots from the same simulated data based upon different numbers of detected genes from 15 (top right) to 240 (bottom right). Download FIG S9, TIF file, 0.6 MB. Copyright © 2023 Homberger et al. 2023 Homberger et al. https://creativecommons.org/licenses/by/4.0/ This content is distributed under the terms of the Creative Commons Attribution 4.0 International license.

We also explored how PCA clustering is affected by the number of detected genes. For the four growth conditions tested, a minimum of 80 detected genes per cell led to clustering results qualitatively similar to those obtained using a much larger number of detected genes (Fig. S9B). Our expectation was that a greater number of detected genes would reveal more distinct subpopulations, but this did not appear to be the case within our experimental conditions, where a range between 80 and 126 genes appeared to be sufficient to discriminate between growth phases. This suggests that it may be possible to investigate the structure of expression within a population with as few as tens of thousands of reads per cell given the efficiencies of cDNA conversion and rRNA depletion we have achieved in this study.

## DISCUSSION

Here, we report substantial improvements to our previously established bacterial MATQ-seq protocol (11). Specifically, we focused on three elements of the workflow: (i) integration of automation and minimization of reaction volumes during different steps of the protocol, as well as optimization of the bioinformatic pipeline for data analysis; (ii) selection of a more efficient RT; and (iii) implementation of an rRNA depletion step by integrating DASH into the library preparation. We validated our improved MATQ-seq protocol by generating a large data set of single Salmonella cells sampled over different growth conditions. Overall, our data show that the changes we implemented increased the cell throughput and robustness of the protocol while reducing cell loss. In addition, we were able to improve gene coverage and the gene detection limits. We were even able to detect sRNAs at the single-cell level, which previously had not been feasible. This will allow the exploration of the regulatory functions of sRNA at the single-cell level in future studies. Moreover, our data confirm previously described heterogeneity within the same cell population, especially regarding Salmonella pathogenicity genes and genes encoding components of the flagellum (32, 34).

The successful implementation of DASH to deplete rRNA-derived cDNA was instrumental in achieving these advances. We believe that DASH can be adapted to other single-cell approaches, which currently do not include a targeted rRNA depletion protocol (11, 14, 15). Because depletion is performed at the cDNA level, DASH only needs to be customized to the library preparation step. For protocols that also use Nextera XT for library preparation, such as PETRI-seq (15), a direct application without any further adjustments is possible. The implementation of DASH would reduce the required sequencing depth and overall sequencing costs.

A general limitation of current scRNA-seq workflows is efficient cell lysis, which might require species-specific customization. Consequently, analysis of mixed bacterial communities is a challenge. This is especially true if their cell wall compositions vary, as this necessitates different enzymatic disruption and poses the risk of introducing bias based on varying lysis efficiency. Combinatorial indexing-based protocols (14, 15) can cope better with this limitation than MATQ-seq, because these protocols can process many more cells at once. Inefficient lysis is therefore compensated for by a higher number of input cells, although the danger of introducing bias remains. In contrast, MATQ-seq is more limited in throughput because of the cell sorting step and therefore inefficient lysis will lead to a high rate of failure. Nevertheless, MATQ-seq can in principle be applied to mixed cell populations if lysis conditions can be optimized.

Due to the high transcript capture rate of MATQ-seq, this method is particularly well suited for experimental settings in which the starting material is limited, such as the analysis of small subpopulations of bacterial cells in host niches or intracellular bacteria. In these settings, the application of dual RNA-seq allows the study of host-pathogen interactions through the simultaneous analysis of the transcriptomes of both the bacteria and the host (1, 35). Single-cell dual RNA-seq (scDual-Seq) has been attempted, but so far, either bacterial gene detection has been inefficient (36) or the experiments were performed under a high multiplicity of infection, which does not reflect physiological conditions (37). Since MATQ-seq was initially developed for scRNA-seq in eukaryotes (12), we see high potential in establishing scDual-Seq with MATQ-seq to capture both eukaryotic and prokaryotic transcripts. In this context, it is interesting that DASH has been shown to remove bacterial as well as eukaryotic rRNA at a single-cell level (22). Nevertheless, establishment of an scDual-Seq protocol based on MATQ-Seq will require further testing and validation.

## MATERIALS AND METHODS

### Bacterial strains and growth conditions.

The bacterial strains used in this study are listed in Table S1E. Salmonella enterica serovar Typhimurium SL1344 (38) and constitutively green fluorescent protein (GFP)-expressing strain SL1344 (39) were grown in Lennox broth (LB) (tryptone [10 g/L], yeast extract [5 g/L], and sodium chloride [85.6 mM]) medium. The GFP-expressing Salmonella strain was used only for FACS gating. Bacterial cultures were inoculated to an optical density (OD) at 600 nm of 0.01 and incubated at 37°C with agitation (220 rpm) until early exponential, mid-exponential, late exponential, and early stationary phases (ODs of 0.1, 0.3, 1.0, and 2.0; according to reference 31). A 1 mL volume of each culture was pelleted and washed twice with 1 mL of 1× Dulbecco’s phosphate-buffered saline (DPBS). Afterwards, the pellet was resuspended in 1 mL of 100% RNAlater/RNAprotect tissue reagent (Qiagen) and kept on ice. Right before sorting, samples were diluted 1:20 in 1× DPBS.

All pipetting steps in the sections below were automated using an I.DOT (Dispendix) dispensing robot except for cleanup and quality control steps.

### Isolation of single cells.

Isolation of single cells was done as previously described by Imdahl et al. (11). Briefly, single cells were isolated using a BD FACS Aria III for sorting individual cells into 96-well plates prefilled with lysis buffer (0.26 μL of 10× lysis buffer [TaKaRa], 0.03 μL of RNase inhibitor [100 U/μL; TaKaRa], 0.26 μL of DPBS [Gibco], 0.1 μL of lysozyme [50 U/μL; Epicentre], and 1.95 μL of nuclease-free water [Ambion]). After sorting, plates were kept on ice and stored at −80°C until further processing.

### Improved MATQ-seq protocol.

The improved MATQ-seq protocol is based on the protocol described by Imdahl et al. (11), with several modifications. Briefly, reverse transcription was performed using primers described by Sheng et al. (12). Instead of SuperScript III, SuperScript IV was used (Invitrogen) as a reverse transcriptase without changing RT reaction volumes. As reaction buffer, SS IV buffer was used instead. Reverse transcription was followed by primer digestion, RNA digestion, and poly(C) tailing. Subsequent second-strand synthesis was performed before PCR amplification with only one-fourth of the reaction volume (40 μL instead of 160 μL). cDNA purification was performed using AMPure XP beads (Beckman Coulter) at a 1:1 (vol/vol) ratio. cDNA quality was checked by using Qubit Flex and the 2100 Bioanalyzer DNA High Sensitivity kit (Agilent Technologies). Oligonucleotides used for MATQ-seq are listed in Table S1F.

### DASH sgRNA pool generation.

The sgRNA pool for Cas9-based ribosomal depletion in Salmonella was generated according to Prezza et al. (21). Briefly, a double-stranded DNA (dsDNA) template for *in vitro* transcription was generated with the oligonucleotide pool composed of 797 sequences targeting rRNA of Salmonella (JVO-21893 [Table S1F]) as well as the fill-in reaction oligonucleotide (JVO-21894 [Table S1F]) using KAPA HiFi HotStart ReadyMix (Roche). After column-based cleanup and quality control on Nanodrop and the Bioanalyzer DNA 1000 kit (Agilent), the sgRNA pool was generated by *in vitro* transcription using MEGAshortscript T7 transcription kit (Invitrogen). The final pool, consisting of 797 sgRNAs, was purified using the Monarch RNA cleanup kit (500 μg; New England BioLabs [NEB]). sgRNA quality control was performed with Qubit and the Bioanalyzer RNA 6000 Pico kit (Agilent).

### Single-cell RNA-seq: library preparation, including ribosomal depletion.

cDNA obtained from single cells after MATQ-seq was further processed for library preparation, including a ribosomal depletion protocol. Library preparation was done using the Nextera XT DNA library preparation kit (Illumina) including the DASH protocol (according to reference 21, with several modifications) with only one-fourth of overall reaction volumes compared to the manufacturer’s recommendations. Tagmentation was performed with 5 μL of total reaction volume instead of 20 μL and 0.5 ng of cDNA input. Index PCR was performed with 13 cycles using Integrated DNA Technologies (IDT) for Illumina Nextera DNA unique dual indexes and a total reaction volume of 12.5 μL instead of 50 μL (Illumina). Obtained libraries were purified with AMPure XP beads (Beckman Coulter) at a 1:1 (vol/vol) ratio to ensure capture of sRNA-derived cDNA. After quality control, up to 12 samples were pooled equimolar for ribosomal depletion. sgRNA/Cas9 complex formation was followed by DASH using the appropriate ratio of cDNA, Cas9, and sgRNA. Cas9 enzyme was inactivated by proteinase K (15 min at 37°C). Afterwards, proteinase K was inactivated by adding PMSF (1 mM final concentration). Depleted cDNA was purified by another round of AMPure XP bead cleanup and used as the input for a second PCR amplification. Depleted cDNA (0.5 ng) was used as the input for Nextera XT reactions omitting the tagmentation steps. Index-independent primers i5 and i7 were used to amplify noncleaved cDNA products. PCR was done for 13 cycles, and cleanup was performed with AMPure XP beads at a 1:1 (vol/vol) ratio. Oligonucleotides used for the second PCR are listed in Table S1F.

### Total RNA extraction and library preparation.

Bacterial RNA was isolated from Salmonella strain SL1344 grown under the same conditions as for scRNA-seq experiments. RNA extraction was performed with 1.8 mL of each *in vitro* culture using the TRIzol reagent (Invitrogen) according to the manufacturer’s recommendation. RNA quality was checked using the Qubit RNA high-sensitivity assay kit (Invitrogen) and 2100 Bioanalyzer RNA 6000 Pico/Nano kit (Agilent Technologies). Prior to library preparation, DNase treatment was performed using a DNase I kit (Thermo Fisher), followed by rRNA depletion. rRNA was depleted using Lexogen’s RiboCop META rRNA depletion kit protocol according to the manufacturer’s recommendation using 100 ng of total RNA as the input per sample. DNA libraries suitable for sequencing were prepared using the CORALL total RNA-Seq library prep protocol (Lexogen) according to the manufacturer’s recommendation with 13 PCR cycles. Library quality was checked using a 2100 Bioanalyzer DNA High Sensitivity kit.

### Sequencing.

Sequencing pools of single cells as well as total RNA-seq libraries were checked using the Qubit DNA High Sensitivity assay kit and a 2100 Bioanalyzer DNA High Sensitivity kit. Sequencing of library pools, spiked with 1% PhiX control library, was performed in single-end 100-cycle sequencing mode on the NextSeq 2000 or NovaSeq 6000 platform (Illumina). Demultiplexed FASTQ files were generated with bcl2fastq2 v2.20.0.422 (Illumina).

### Bioinformatics.

**(i) Preprocessing.** Read trimming and quality control of MATQ-seq reads were executed using BBDuk (40) and MultiQC (41). To efficiently remove primer and adapter sequences located at both ends of a read, we ran BBDuk in a two-pass procedure using the default adapter sequence database augmented with MATQ-seq-specific sequences (Table S1G). The first pass focused on the 5′ end, with parameters *minlen=18 qtrim=rl trimq=20 ktrim=l k=17 mink=11 hdist=1 trimpolya=30*, while the second pass focused on the 3′ end, with parameters *minlen=18 qtrim=rl trimq=20 ktrim=r k=17 mink=11 hdist=1*.

**(ii) Read alignment and counting.** Read alignment and counting were performed with Bowtie2 (42) and featureCounts (43), allowing a single mismatch and run-in --*local mode*. BigWig files were generated using deepTools (44), passing additional parameters --*binSize 5* --*normalizeUsing BPM*. We employed the same gene detection method from the original MATQ-seq analyses (11), requiring a detected gene to have >5 reads.

**(iii) Normalization and differential expression.** DESeq2 (45) was used for normalization and differential expression analysis, using size factors calculated by the *computeSumFactors* function in Scran (46) and other recommended parameters for DESeq2 single-cell analysis, which included using the likelihood ratio test (LRT), *useT=TRUE*, *minmu=1e-6*, and *minReplicatesForReplace=Inf*.

**(iv) Identification of outlier cells.** To identify outlier cells, we calculated the average number of detected genes per cell (>5 reads) for each condition. Cells were determined to be outliers if their detected gene number varied more than 2 standard deviations (SD) above or below the mean, which removed 14 cells. One additional cell was removed based on the PCA plot generated using PCAtools (47).

**(v) Comparison with existing bulk and single-cell RNA-seq data.** To ensure fair comparisons between bulk RNA-seq and scRNA-seq data sets, we processed all FASTQ files using the same preprocessing, alignment, and counting approaches as described above. For our pseudo-bulk representation from the single cell data, for each condition, we summed the counts across all cells for each gene. Bulk RNA-seq data were generated in parallel with the single-cell data.

**(vi) Small RNAs and highly variable genes.** Additional small RNAs (sRNAs) were added to our annotation, giving us 172 sRNAs in total. To show the most abundant sRNA in the heat map for Fig. 5, sRNAs were only shown if the row sums of Transcripts Per Million (TPM) normalized counts across all conditions was >100. The full list of expressed sRNAs is provided in Table S1D. Highly variable genes were identified using Scran (46), with the top 1% of HVG used for the heat map in Fig. 6. The number of Salmonella pathogenicity and flagellar genes used in the supplementary heat maps (Fig. S7 and S8) was reduced to show only genes expressed under the examined conditions.

**(vii) Single-cell simulations and downsampling.** Simulated data were generated using the *sample* function in R. All Salmonella genes (including rRNA) were sampled with replacement, with read count frequencies used as probability weights per cell for each condition. Different sample sizes were used to represent sequencing read depth, and detected genes were the resulting uniquely sampled genes.

### Data availability.

Data are available under Gene Expression Omnibus (GEO) accession number GSE218633. Bioinformatic scripts used for analysis are available on GitHub: https://github.com/BarquistLab/MATQ-seq_2023.
