# Supplementary figures and images for: Improved Bacterial Single-Cell RNA-Seq through Automated MATQ-Seq and Cas9-Based Removal of rRNA Reads
Source: mBio. 2023 Mar 7;14(2):e03557-22. doi: 10.1128/mbio.03557-22 (PMC10127585; doi:10.1128/mbio.03557-22)

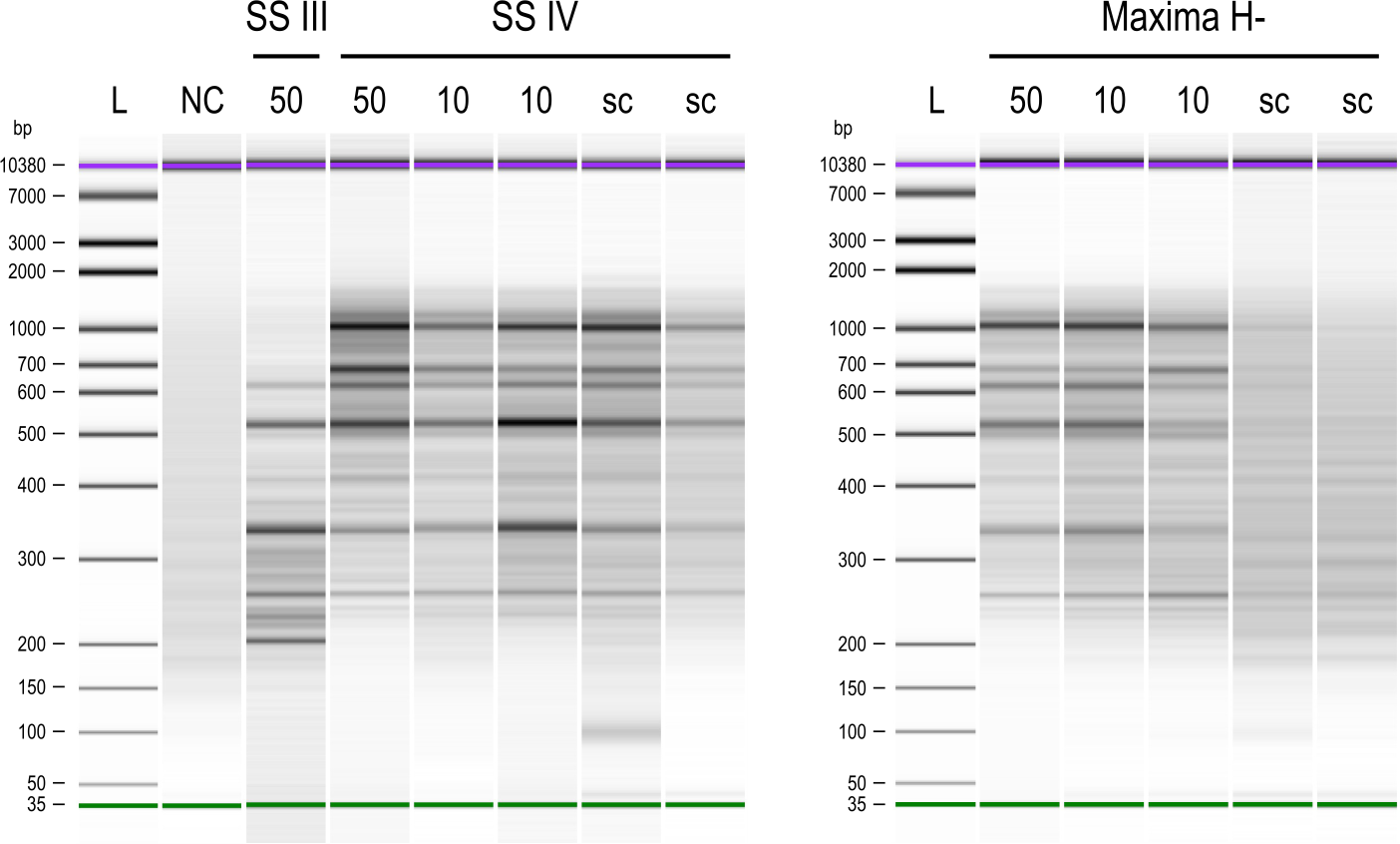

Supplement: FIG S1 [file mbio.03557-22-s0002.tif]

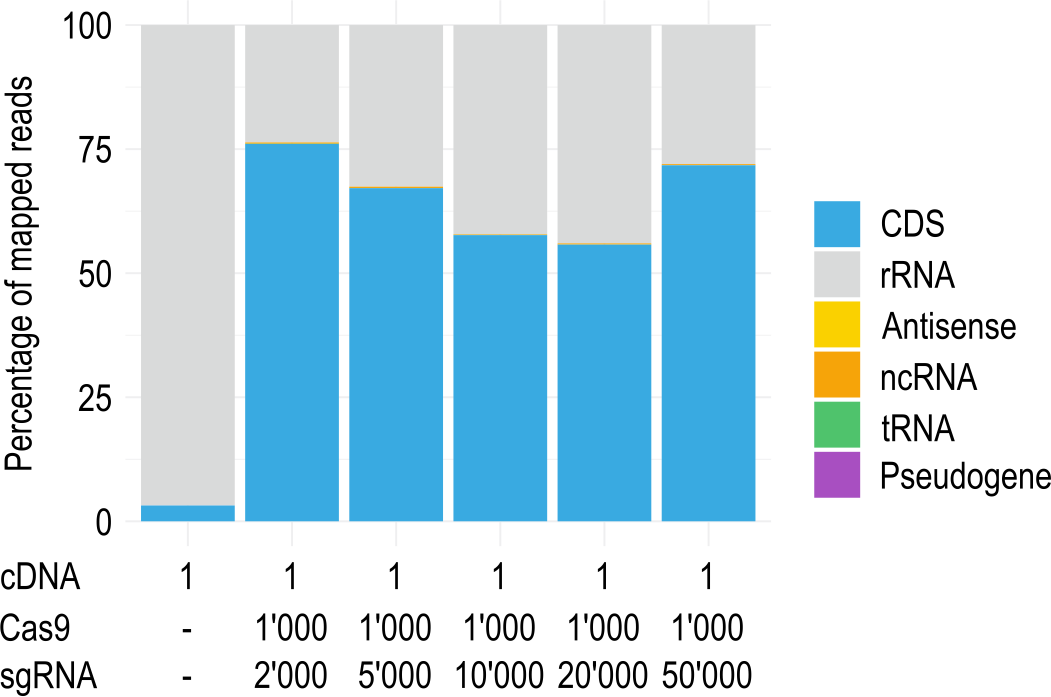

Supplement: FIG S2 [file mbio.03557-22-s0003.tif]

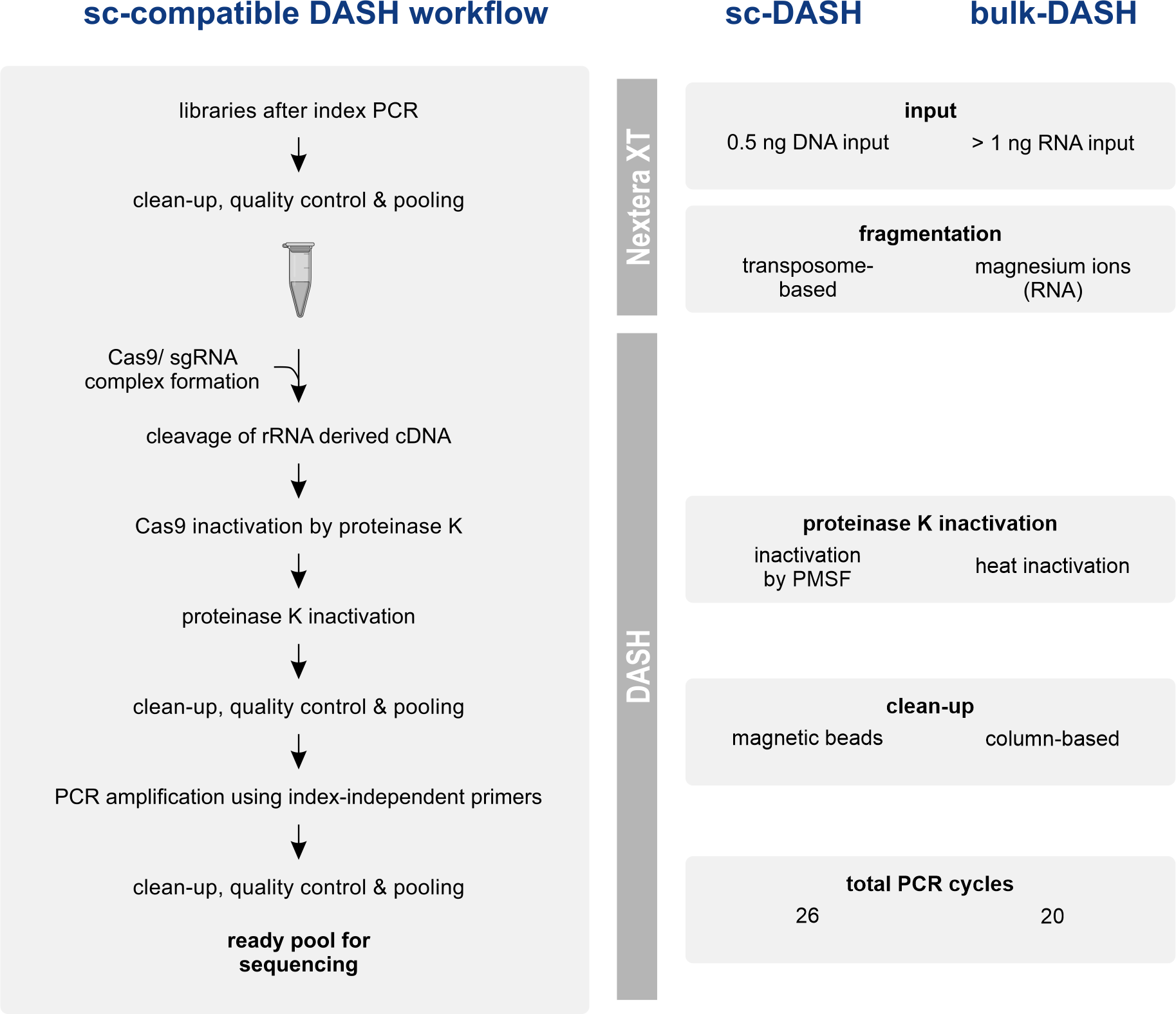

Supplement: FIG S3 [file mbio.03557-22-s0004.tif]

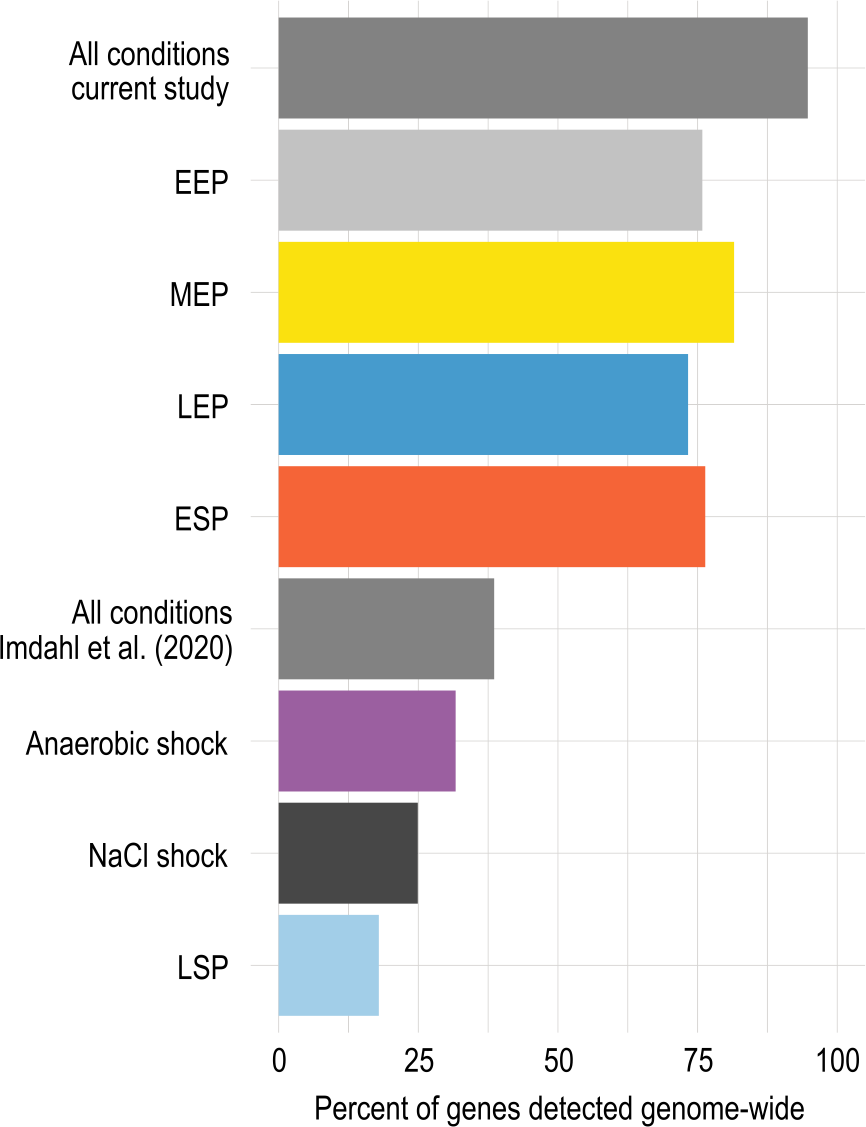

Supplement: FIG S4 [file mbio.03557-22-s0005.tif]

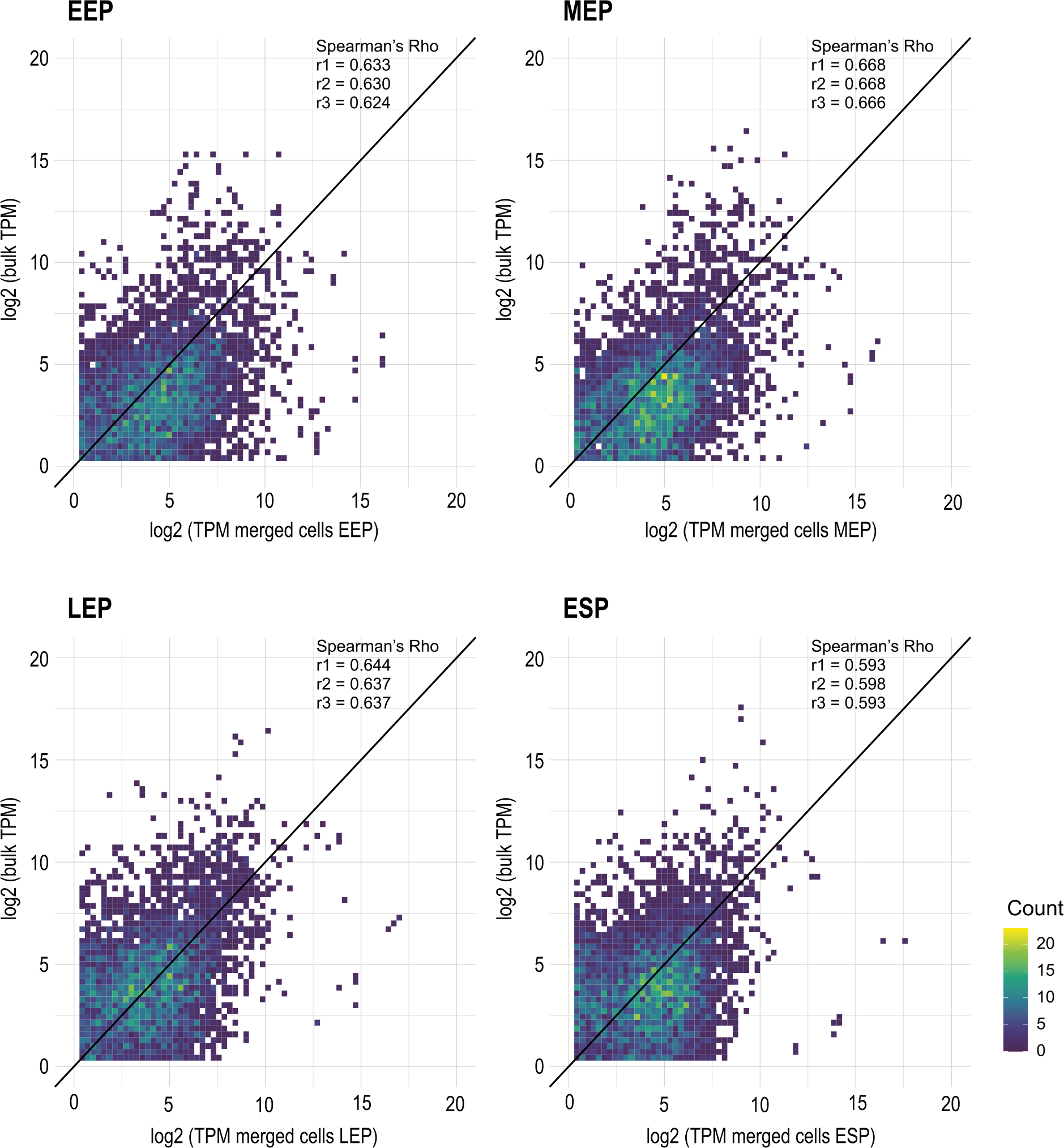

Supplement: FIG S5 [file mbio.03557-22-s0006.tif]

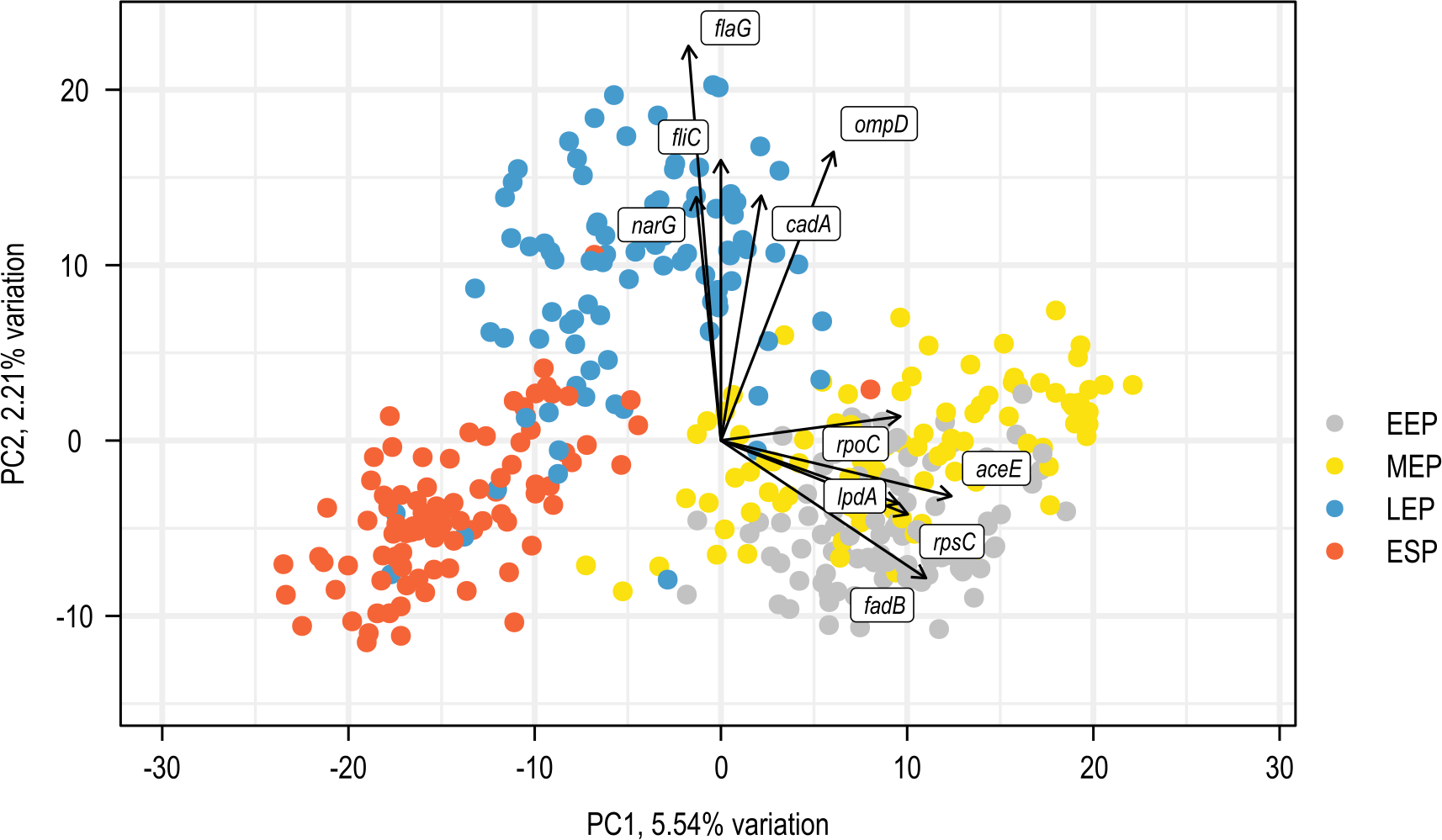

Supplement: FIG S6 [file mbio.03557-22-s0007.tif]

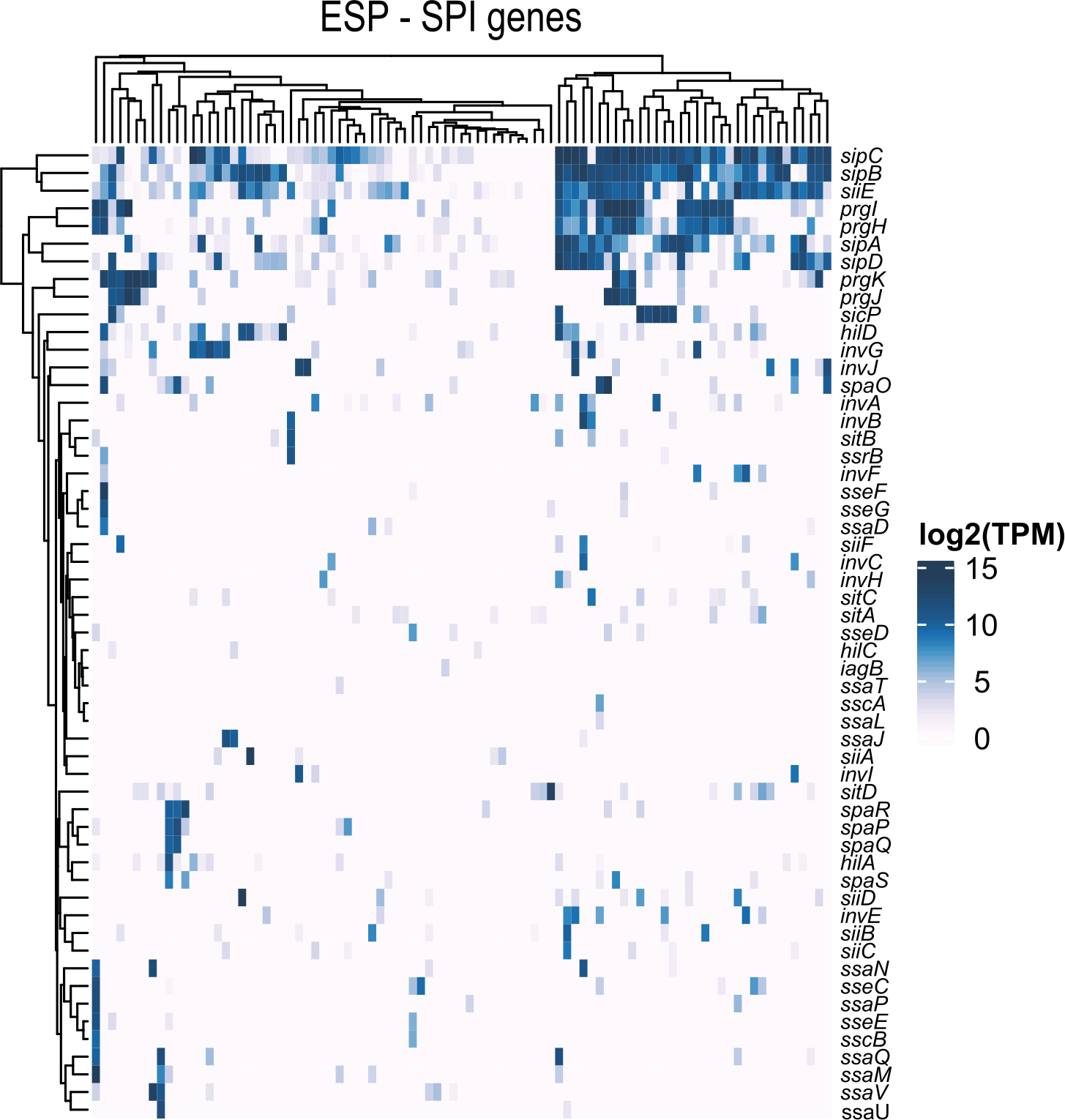

Supplement: FIG S7 [file mbio.03557-22-s0008.tif]

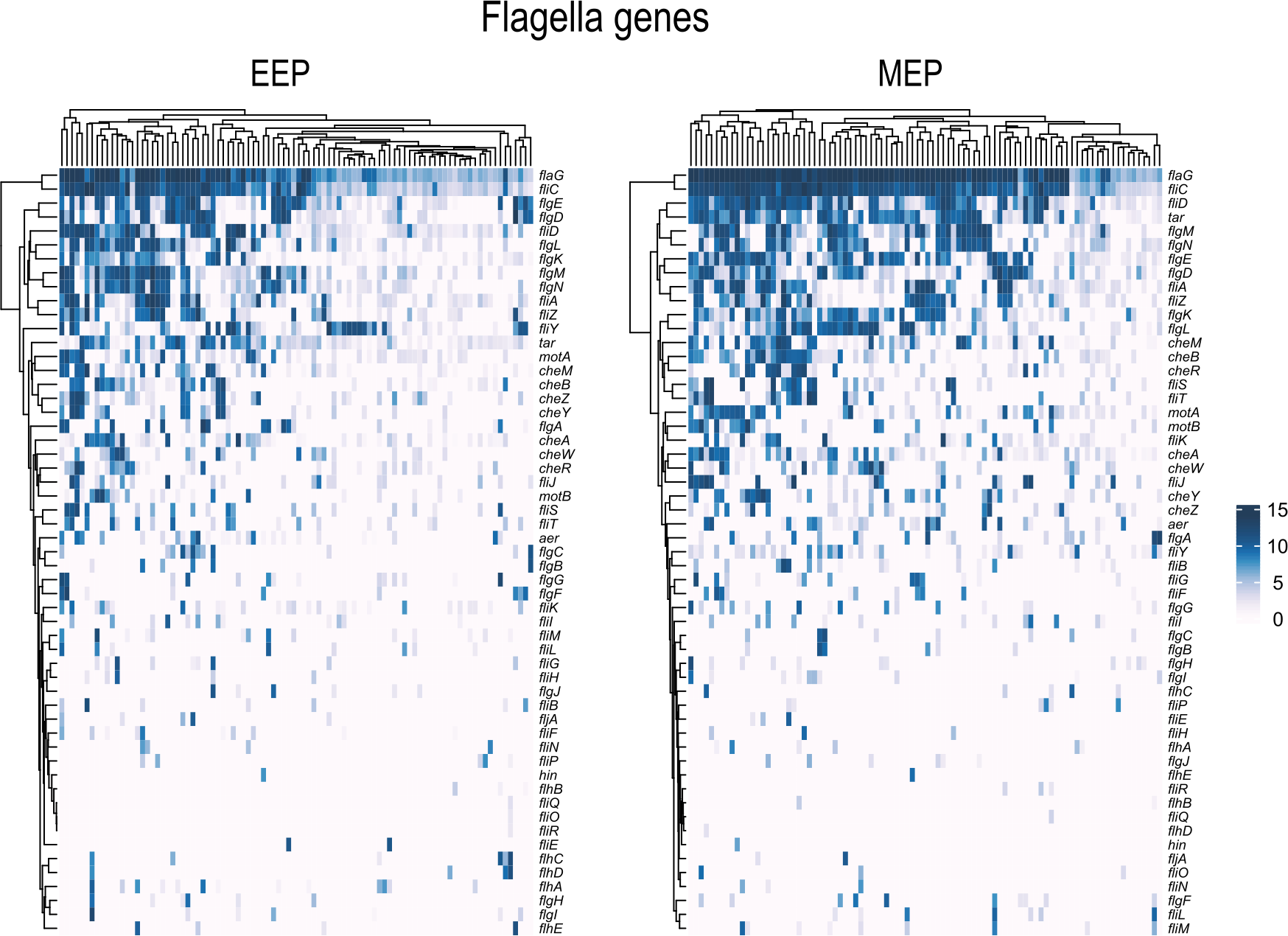

Supplement: FIG S8 [file mbio.03557-22-s0009.tif]

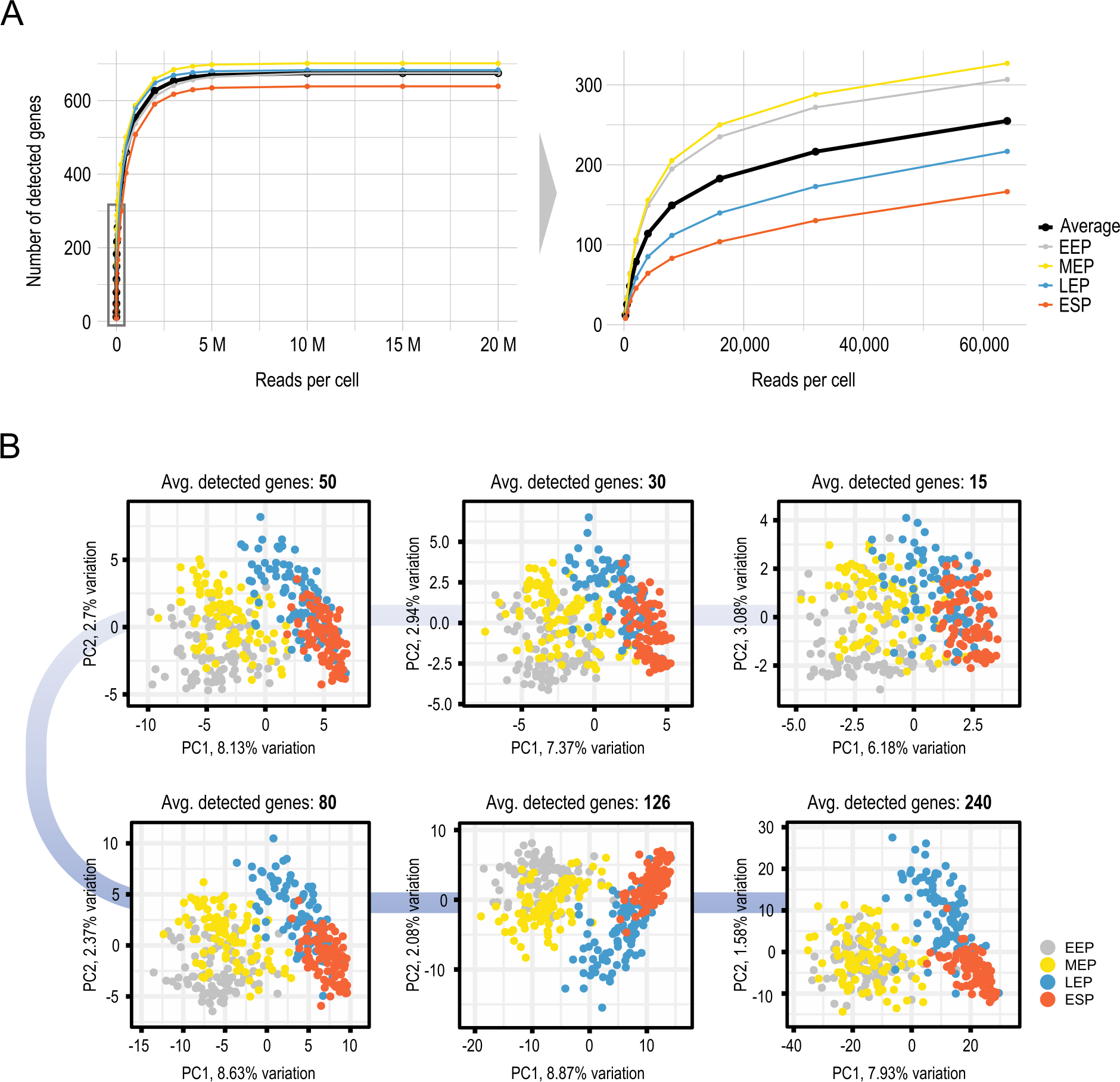

Supplement: FIG S9 [file mbio.03557-22-s0010.tif]
